# Validation of a deep neural network-based algorithm supporting clinical management of adnexal mass

**DOI:** 10.3389/fmed.2023.1102437

**Published:** 2023-01-23

**Authors:** Gerard P. Reilly, Charles J. Dunton, Rowan G. Bullock, Daniel R. Ure, Herbert Fritsche, Srinka Ghosh, Todd C. Pappas, Ryan T. Phan

**Affiliations:** ^1^Axia Women’s Health, Cincinnati, OH, United States; ^2^Aspira Women’s Health, Austin, TX, United States; ^3^ICON plc, Portland, OR, United States

**Keywords:** conservative management, benign ovarian, ovarian, malignancy, cancer, pelvic mass

## Abstract

**Background:**

Conservative management of adnexal mass is warranted when there is imaging-based and clinical evidence of benign characteristics. Malignancy risk is, however, a concern due to the mortality rate of ovarian cancer. Malignancy occurs in 10–15% of adnexal masses that go to surgery, whereas the rate of malignancy is much lower in masses clinically characterized as benign or indeterminate. Additional diagnostic tests could assist conservative management of these patients. Here we report the clinical validation of OvaWatch, a multivariate index assay, with real-world evidence of performance that supports conservative management of adnexal masses.

**Methods:**

OvaWatch utilizes a previously characterized neural network-based algorithm combining serum biomarkers and clinical covariates and was used to examine malignancy risk in prospective and retrospective samples of patients with an adnexal mass. Retrospective data sets were assembled from previous studies using patients who had adnexal mass and were scheduled for surgery. The prospective study was a multi-center trial of women with adnexal mass as identified on clinical examination and indeterminate or asymptomatic by imaging. The performance to detect ovarian malignancy was evaluated at a previously validated score threshold.

**Results:**

In retrospective, low prevalence (*N* = 1,453, 1.5% malignancy rate) data from patients that received an independent physician assessment of benign, OvaWatch has a sensitivity of 81.8% [95% confidence interval (CI) 65.1–92.7] for identifying a histologically confirmed malignancy, and a negative predictive value (NPV) of 99.7%. OvaWatch identified 18/22 malignancies missed by physician assessment. A prospective data set had 501 patients where 106 patients with adnexal mass went for surgery. The prevalence was 2% (10 malignancies). The sensitivity of OvaWatch for malignancy was 40% (95% CI: 16.8–68.7%), and the specificity was 87% (95% CI: 83.7–89.7) when patients were included in the analysis who did not go to surgery and were evaluated as benign. The NPV remained 98.6% (95% CI: 97.0–99.4%). An independent analysis set with a high prevalence (45.8%) the NPV value was 87.8% (95% CI: 95% CI: 75.8–94.3%).

**Conclusion:**

OvaWatch demonstrated high NPV across diverse data sets and promises utility as an effective diagnostic test supporting management of suspected benign or indeterminate mass to safely decrease or delay unnecessary surgeries.

## 1. Introduction

Adnexal masses present a common diagnostic challenge to obstetricians/gynecologists. Up to 10% of women will undergo surgical intervention for an adnexal mass during their lifetime (1), for an adnexal mass which is usually discovered with imaging, either incidentally or due to symptoms. The true incidence of these masses is impossible to determine, but is higher than the number that requires surgery, since many asymptomatic masses are never discovered, or spontaneously resolve (2–4).

Ovarian cancer is aggressive, with an estimated 5-year relative survival rate of less than 50% (5). Therefore, estimating the risk of malignancy of an adnexal mass is the highest concern in management. Ovarian malignancy is, however, rare, even among women with an adnexal mass. In so-called “simple cystic masses” the rate is likely below 3% (2, 6, 7). A recent large-scale ovarian cancer screening trial found that in women with initial abnormal transvaginal ultrasonogram (TVUS) findings, over 60% of the masses resolved on subsequent US (8). The IOTA5 study showed a 20% spontaneous resolution rate, with very low (<1%) rates of complications such as rupture, torsion, or malignant transformation (9). These data suggest that conservative management of a mass should be considered, especially where physicians and patients want to avoid or delay surgery. Imaging has been shown to be generally good at identifying benign masses (7–9). Various studies, however, have reported that imaging might miss malignancy in these patients (1, 10). Additional risk assessment tools, including biomarkers, and clinical algorithms utilizing and multivariate classifiers, could help physicians identify masses unlikely to be malignant. This would reduce unnecessary surgeries and associated complications.

Multivariate index assay (MIA) and MIA2G were developed for patients who have an adnexal mass and are scheduled for surgery (10–12). The function of these FDA-cleared tests is to determine if the patient should be referred to a gynecologic oncologist for management, or if the risk of malignancy is low enough to allow a gynecologic generalist to manage the patient. Today, there is no clinical testing available to assess risk in suspected benign and indeterminate masses. In a previous publication we described the development of a proprietary machine learning-based classification model, MIA3G, to determine the risk of malignancy in patients who had presented with an adnexal mass. This model utilizes a set of seven biomarkers and the patient’s age and menopause status (13). In a large retrospective cohort of over 2,000 patients with a prevalence of 4.9%, MIA3G showed 90% sensitivity and 84% specificity for identifying ovarian malignancy. MIA3G achieved sensitivities of 94.9% for epithelial ovarian cancer, 76.9% for early-stage cancer, and 98.0% for late-stage cancer. MIA3G also showed a 99.4% negative predictive value (NPV), indicating the utility of this test for conservative management of adnexal masses. Utilizing MIA3G model, we report the development and performance characteristics of OvaWatch using a prospective cohort of 546 patients presented with adnexal masses. OvaWatch is the first risk assessment tool supporting clinicians to make informed decisions to manage patients with an adnexal mass with initial clinical assessment as benign or indeterminate.

## 2. Materials and methods

### 2.1. OvaWatch algorithm development and description

MIA3G is a proprietary deep feed-forward neural network (DNN)-based algorithm developed with the aim: *low* and *elevated* risk of malignancy. Data from a heterogenous set of 3,067 patient samples from previous clinical studies (12, 14, 15) were randomly assigned to training/testing and validation sets to derive and characterized the performance of the algorithm. All patients had undergone to surgery and thus had pathology confirmation of benign or malignant adnexal mass. The sample size of the malignant and benign cohorts was further balanced for algorithm training using a modification of the synthetic minority oversampling (SMOTE) (13). The following features: age, menopausal status, and seven protein biomarker measurements were trained *via* a neural network to known histopathological diagnoses of ovarian malignancy (malignant vs. non-malignant) as the labels. Seven biomarkers used are cancer antigen 125 (CA125), human epididymis protein 4 (HE4), beta-2 microglobulin (B2M), apolipoprotein A-1 (ApoA1), transferrin (TRF), Prealbumin, (PreAlb), and follicle-stimulating hormone (FSH). MIA3G algorithm utilized multiple hidden layers each with their own weighted nodes and activation functions (16). The neural network is regularized using node dropout to reduce overfitting where a percentage of the nodes are randomly omitted from each hidden layer during training (17). The final layer of the neural network had two nodes and uses the *softmax* function to assign the probability of binary classification as *low* or *elevated* risk of malignancy. Further details of the classifier development have been previously described (13).

The OvaWatch test score was derived from the MIA3G algorithm. It was calculated as the softmax probability of elevated risk of malignancy scaled by 10, rounded down using a “floor” function and binning into units of 0.5. For this report, we apply the validated threshold value of a MIA3G softmax-high score of 0.5 (OvaWatch score of 5.0) from our previous study (13).

### 2.2. Nomenclature

To delineate the differences across OvaWatch test, surgical histology, and physicians’ assessment outcomes we adopt the following terminology throughout this report. In the retrospective studies where physicians were required to provide an independent assessment of the adnexal mass, the terms *assessment benign* and *assessment malignant* are used. The results of OvaWatch are labeled as *low risk of malignancy* and *indeterminate* depending on whether the test result is above or below the score threshold, respectively. Note that this contrasts with the terminology used for the parent algorithm (MIA3G) of *low* or *elevated* risk of malignancy. The diagnostic accuracy of physician assessment or OvaWatch was evaluated against the “gold standard” of surgical histology which is referred to as *histologically benign* or *histologically malignant*.

### 2.3. Data and ethics

All data were obtained from adult patients who provided informed consent to participate in the research. All research was carried out under Institutional Review Board (IRB)-approved protocols. Protocol numbers are provided in Table 1.

**TABLE 1:** Enrollment sites and internal and institutional review board (IRB) study identifiers included in the prospective analyses.

Site name/Number	Enrollment	First patient enrolled	Study/IRB#
Axia Women’s Health/01	50	6/25/2020	OVANex/08-2020
May Grant OBGYN/04	110	10/21/2020	OVANex/08-2020
Hill Country OBGYN/05	94	1/21/2021	OVANex/08-2020
Square Medical OBGYN/06	68	12/8/2020	OVANex/08-2020
Premier OBGYN/09	44	3/19/2021	OVANex/08-2020
MidTown OBGYN/10	33	9/23/2021	OVANex/08-2020
Women’s Health of Mobile/11	6	3/23/2022	OVANex/08-2020
New Horizon’s Clinical Trials/12	4	5/17/2022	OVANex/08-2020
Altus Research/03	130	9/21/2020	OVANex/04-2019
Northwell Health/08	82	7/13/2021	OVANex/04-2019
New Horizon’s Clinical Trials/04	96	1/19/2021	OVANex/05-2020

### 2.4. Studies and sample sets

This study presents validation of datasets–both retrospective and prospective–from multiple studies spanning multiple centers. Broadly, the inclusion criteria for these studies were as follows: (1) Patient age ≥ 18 years, (2) informed consent provided by the patient to participate in research, (3) patient agreeable to phlebotomy, (4) patient had a documented adnexal mass. The adnexal mass was confirmed by imaging (CT, TVUS, or MRI) prior to enrollment. In the retrospective studies, all patients were scheduled for surgical intervention within 3 months of imaging. Exclusion criteria included a diagnosis of malignancy in the previous 5 years (except non-melanoma skin cancers). Exclusion criteria also included adnexal surgery within 6 weeks prior to enrollment in the study.

Retrospective studies had previously been used to develop and validate MIA, MIA2G (12, 14), and MIA3G (13). Because these data sets had information on physicians’ independent clinical assessment of the malignancy of the mass, consistent with the intended uses of MIA and MIA2G, it was possible to stratify patients based on this assessment. Data from the assessment of benign patients comprised the “Multivariate Index Assay Benign” (MIAB) dataset and is further described in the “3. Results” section.

The validation included samples from ongoing prospective studies (Table 1), which is referred to in this report as the “prospective real-world” (PRW) study and described further in the “3. Results” section. Data and sample collection protocols were identical for all samples. The subjects had a documented adnexal mass and were not yet scheduled for surgery. Patients were stratified on enrollment into cohorts A, B, or C based on physician determination. Cohort A comprised patients who had a mass and were symptomatic with symptoms such as pelvic pain, bloating or frequent urination and, as per physician’s assessment, signs of potential malignancy on imaging, for example: complex cyst, solid mass, ascites. Cohort B comprised patients who were asymptomatic but discovered to have adnexal mass on exam or imaging. Cohort C consisted of those with known genetic risk or family history of ovarian cancer, and were permitted enrollment without an adnexal mass, although only patients with a documented adnexal mass from this cohort were included in this analysis.

For patients who did not immediately go to surgery within the period of this study, there may have been multiple blood draws to follow changes in biomarkers. Data from follow-up draws have not been included in this report. Blood was drawn at the time of enrollment and batch-tested asynchronously for biomarkers and OvaWatch test score determination. The physician was not provided OvaWatch results at any point in the trial. At the physician’s request, they could receive either CA125 results or MIA results to augment clinical decision-making.

A high-prevalence “independent assessment” (IA) set was assembled using a combination of (1) benign samples from the three prospective studies mentioned in Table 1, and (2) commercially-sourced serum samples from Accio Biobank Online (SHARE Bio-repository, Spectrum Health Network) and USBioLab (Fox Chase Cancer Center) These samples were obtained from patients with a documented adnexal mass which was planned for surgical intervention within 3 months of imaging (CT, TVUS, or MRI).

### 2.5. Determination of serum biomarker values

The serum biomarker values for the prospective studies RP-08-2020, RP-09-2020, RP05-2019 were generated and run at a CAP-accredited CLIA laboratory (Aspira Labs, Austin TX, USA). For patients in these protocols, a pre-operative blood sample of approximately 8.5 ml was collected into a serum processing tube and separated with centrifugation within 1–6 h of collection. The sample was stored at 2–8 degrees C and shipped to the laboratory on wet ice within 8 d of collection. All serum biomarker concentrations were determined on the Roche cobas 6,000 clinical analyzer, utilizing the c501 and e601 modules and Roche Diagnostics’ clinical assays. Biomarkers were run using assays that had passed rigorous lot acceptance criteria per laboratory QA/QC procedures. All measurements were performed on coded samples (blinded to patient demographics and/or pathology outcome).

### 2.6. Statistics and data analysis

We evaluated the statistical powering of the prospective clinical study over a range of prevalences assuming a sample size of 546 and a hyper-geometric distribution, i.e., no resampling in the main population of patients being evaluated. At a confidence level of 95% and a power of 80% we would need to observe histological pathology results from 28 patients at 10% prevalence, 56 patients at 5% prevalence, or and 143 patients at 1.8% prevalence.

OvaWatch scores for all patient cases were generated in the R Statistical Programming Language (ver 4.2.1) (18) using Tensorflow through the Keras interface (ver 2.4.0). The performance of OvaWatch on the validation cohorts was also performed in R Statistical using the epiR library (ver 2.0.50) to generate estimates and confidence intervals of the binomial statistics. Confidence intervals were generated using Wilson’s method (19). PPV and NPV as a function of prevalence were calculated using the following formulae:


(1) $$\mathrm{PPV}=\frac{\mathrm{Sensitivity}\times \mathrm{Prevalence}}{\mathrm{Sensitivity}\times \mathrm{Prevalence}+\left(1-\mathrm{Specificity}\times \left(1-\mathrm{Prevalence}\right)\right)}$$

(2) $$\mathrm{NPV}=\frac{\mathrm{Specificity}\times \left(1-\mathrm{Prevalence}\right)}{\left(1-\mathrm{Sensitivity}\right)\times \mathrm{Prevalence}+\mathrm{Specificity}\times \left(1-\mathrm{Prevalence}\right)}$$

Bootstrapping approach employed to estimate performance as a function of prevalence was performed in R using the sample_slice (replacement = TRUE) function of the *dplyr* library (ver 1.0.10). A total of 5,000 samples was generated to titrate prevalences from 1 to 10% malignancies. Principal component analysis was performed using prcomp from the base *stats* package of the R and visualized using the *factoextra* library (ver 1.0.7).

## 3. Results

### 3.1. Performance of OvaWatch in retrospective cases independently determined as benign by physicians

**FIGURE 1:**
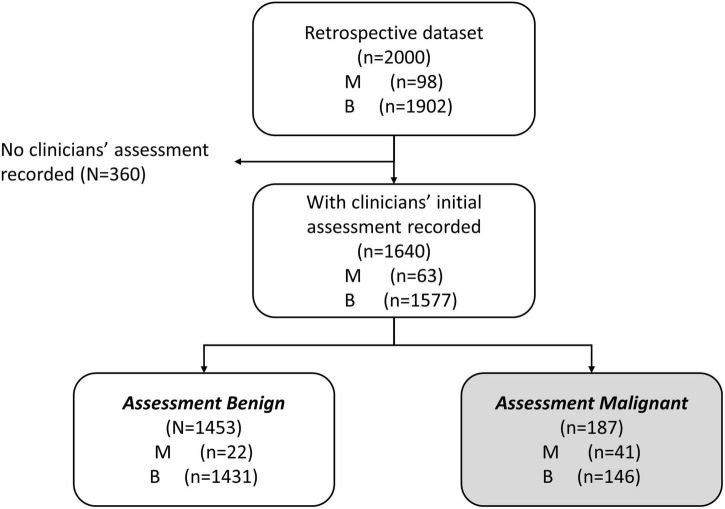
Work-flow diagram showing analysis data set stratified by physician assessment of malignancy risk. A comprehensive retrospective validation was performed on 2,000 samples with 98 malignant and 1,902 benign specimens [citation]. Within these data, 1,640 received an independent physician clinical assessment–using imaging and other clinical examination–as either benign or malignant. A total of 1,453 patients were independently assessed as benign by physician, prior to surgery (MIAB data set).

We were interested in the clinical setting where the physician believed the patient’s mass was benign (assessment benign), rather than at risk for malignancy (assessment malignant). In the studies that were used to derive MIA and MIA2G (12, 14), the physicians were required to provide an independent clinical assessment of the malignancy of a mass prior to surgery and subsequent confirmation based on pathology. The physicians were not provided with the result of MIA for these patients but used imaging, physical examination, and other biomarkers (e.g., CA125) to categorize the mass as assessment benign or assessment malignant. We analyzed the subset of patients that were assessment benign prior to surgery to determine the performance of OvaWatch in patients where the physician presumed the patient’s mass to be benign. The workflow diagram for the derivation of retrospective data set of patients who were assessment benign (MIAB data set) is presented in Figure 1. It is important to note that all cases went to surgery and so had surgical pathology confirmation of diagnosis.

**FIGURE 2:**
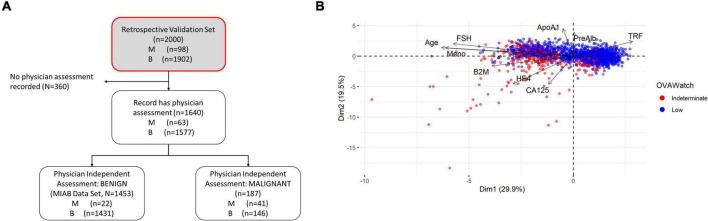
Characteristics of OVAWatch in the retrospective validation set. **(A)** Flow diagram showing patient data represented (shaded, with red outline). **(B)** Principal components analysis visualization bi-plot visualizing of the coordinates of biomarker and clinical variables used in derivation of OVAWatch, and the individual subjects plotted on the first two principal component dimensions. The data set is the original 2,000 patients from the previously published validation. The individual subjects are color coded by their OVAWatch test result (red = indeterminate, blue = low malignancy risk). Abbreviations for biomarkers are in the Methods section, Meno, menopausal status and is a binary variable (pre- or post-menopausal).

**FIGURE 3:**
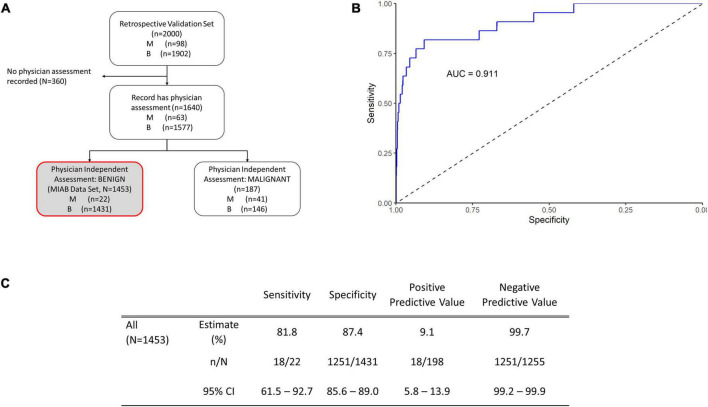
Performance of OVAWatch in the multivariate index assay benign (MIAB) data set. **(A)** Flow diagram showing patient data represented (shaded, with red outline). **(B)** Receiver-Operator Characteristics (ROC) plot of the MIAB data set. The area under the ROC curve (AUC) for OVAWatch was 0.911. **(C)** Performance of OVAWatch in identifying histologically malignant patients in the MIAB data set at an OVAWatch score threshold value of ≥5.0.

Our previous publications showed the performance of OvaWatch, (Sensitivity 89.8% Specificity 84.0% over all cases) (13) in the complete validation data set (prevalence of 4.9% or 98/2000). The influence of the seven biomarkers and clinical features (Age and menopausal status) that contribute to classification of *low probability of malignancy* or *indeterminate* risk are summarized in the plot of the principal components analysis of the entire 2,000 sample validation set (Figure 2). This analysis shows that CA125 and HE4 are positively correlated with classification of indeterminate, whereas TRF and PreAlb are positively correlated with the classification of low probability of malignancy. In the MIAB data set, which comprised 1,453 of 2,000 validation samples, the prevalence was 1.5% (22/1,453). The performance of OvaWatch in the MIAB data set is shown graphically in the Receiver Operator Characteristics (ROC) plot in Figure 3B and is presented for the threshold OvaWatch score of ≥5.0 as indeterminate in the inset table of Figure 3 (Figure 3C).

OvaWatch at a threshold of ≥5.0 had a sensitivity of 81.8.% (95% CI: 61.5–92.7) a specificity of 87.4% (95% CI: 85.6–89.0), and an NPV of 99.7% (99.2–99.9) for detecting histologically malignant patients in this group, as compared to the sensitivity of 89.8% and specificity of 84.0% and a NPV of 99.4% in all evaluated patients, presented previously (13). OvaWatch identified 18 of 22 patients as indeterminate that were not determined as assessment benign by physician assessment alone. Conversely, in patients who were assessment malignant, (187 of 1,640 samples), OvaWatch identified all histologically malignant cases as indeterminate (41/41). OvaWatch had a higher rate of false positives than physician assessment. In assessment benign patients, OvaWatch identified as indeterminate 180 patients who were histologically benign, or a false positive rate of 12.4%. The analysis inclusive of both classes of physician assessment is further detailed in Supplementary Figure 1.

**FIGURE 4:**
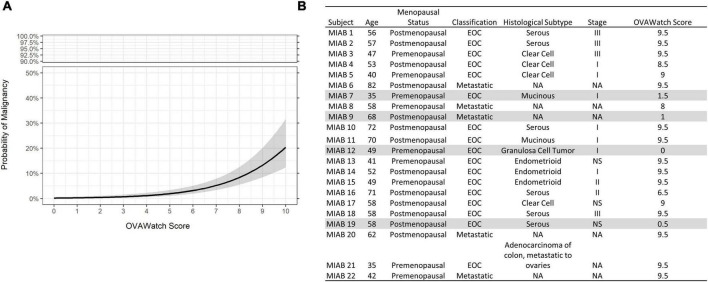
**(A)** Probability of malignancy as a function of OVAWatch in the multivariate index assay benign (MIAB) dataset. **(B)** Table of characteristics of all patients in the MIAB dataset who were histologically malignant. Shaded rows are false negative cases.

The probability of a malignant mass by OvaWatch score in the MIAB data set is shown in Figure 4A. At this low prevalence, this probability is below 5% at the threshold OvaWatch score of 5.0. The inset table (Figure 4B) shows the characteristics of histologically malignant patients in the assessment benign group. The malignancies that OvaWatch called low probability of malignancy are highlighted in gray.

### 3.2. Performance of OvaWatch in a prospective low-prevalence study

**FIGURE 5:**
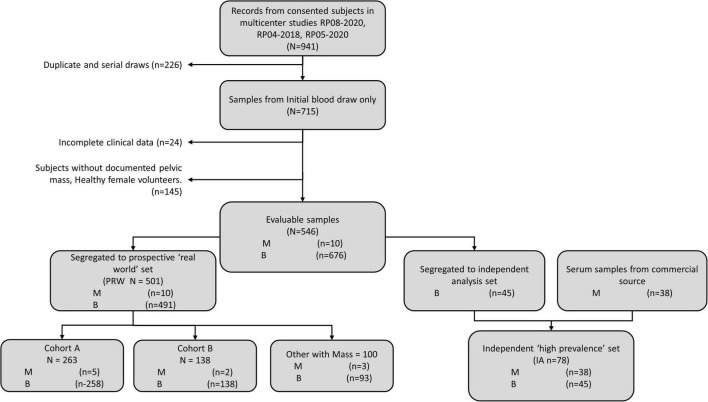
Work-flow diagram showing stratification of samples from prospective studies into the prospective “real world” (PRW) and independent high-prevalence (IHP) data sets.

Validation data were collected in a prospective clinical study of intended-use patients (Table 1, above), but analyzed retrospectively for this study. Physicians did not have access to OvaWatch to support clinical decisions. Some patients received multiple blood draws and tests at suggested intervals throughout the study as part of the protocol, but these exact intervals were determined by the physicians. For this analysis, we only include data from the patient’s initial blood draw and tests. We focused on the first draw only because it would allow the most direct assessment of the test’s sensitivity to detecting malignancy at the first clinical examination opportunity. The flow diagram describing how the data set is comprised is shown in Figure 5. Of 546 evaluable patients in this data set, 151 had surgery for their masses, which exceeds the number of histologically confirmed cases need for proper powering (*n* = 142). The prospective data were further divided into a low-prevalence prospective real world (PRW) validation set and an independent analysis set (IA). The composition of the samples distributed into the data sets is summarized in Table 2.

**TABLE 2:** Prospective study patient demographic and clinical characteristics.

*N*		All		Pre-menopausal		Post-menopausal	
**Individual patients**		**546**		**344**		**202**	
**Mean age**		**47.5**		**41.0**		**58.7**	
**Race and/or Ethnicity**		**N**	**%**	**N**	**%**	**N**	**%**
White/Caucasian		339	62.1%	195	56.7%	144	71.3%
Black or African American		44	8.1%	26	7.6%	18	8.9%
Asian		22	4.0%	20	5.8%	2	1.0%
Hispanic or Latino		19	3.5%	17	4.9%	2	1.0%
Ashkenazi Jewish		1	0.2%	0	0.0%	1	0.5%
Indigenous American or Alaska Native		2	0.4%	2	0.6%	0	0.0%
Native Hawaiian or other Pacific Islander		2	0.4%	1	0.3%	1	0.5%
Other or more than one of the above		76	13.9%	53	15.4%	23	11.4%
Unknown		41	7.5%	30	8.7%	11	5.4%
Non-surgery patients, presumed benign (*n*, %)		395	72.3%	261	75.9%	134	66.3%
Patients with surgical pathology (*n*, %)		151	27.7%	83	24.1%	68	33.7%
**Pathology diagnosis**		**N**	**%**	**N**	**%**	**N**	**%**
Benign ovarian conditions		140	92.7%	77	92.8%	63	92.6%
Low malignant potential (Borderline)		1	0.7%	1	1.2%	0	0.0%
Epithelial ovarian cancer		5	3.3%	2	2.4%	3	4.4%
Non-epithelial primary ovarian cancer		4	2.6%	2	2.4%	2	2.9%
Non-primary malignancies		1	0.7%	1	1.2%	0	0.0%
**Stage (Primary ovarian malignancies)**		**N**	**%**	**N**	**%**	**N**	**%**
Stage I		3	33.3%	1	25.0%	2	40.0%
Stage II		2	22.2%	2	50.0%	0	0.0%
Not Staged		3	33.3%	0	0.0%	3	60.0%
**Histologic subtype (Primary ovarian malignancies)**		**N**	**%**	**N**	**%**	**N**	**%**
Epithelial ovarian cancer (EOC)	Serous	1	11.1%	0	0.0%	1	20.0%
	Endometrioid	1	11.1%	0	0.0%	1	20.0%
	Mixed	1	11.1%	1	25.0%	0	0.0%
Non-EOC	Sex cord stromal (Granulosa cell tumor)	2	22.2%	0	0.0%	2	40.0%
	Sex cord stromal (Sertoli-Leydig tumor)	1	11.1%	1	25.0%	0	0.0%
	Carcinosarcoma	1	11.1%	0	0.0%	1	20.0%
	Leiomyosarcoma	1	11.1%	1	25.0%	0	0.0%

The PRW data set had a prevalence of 9.4% (10/106) when considering only histologically confirmed malignancies, and 2.0% (10/501) when considering all first-draw patients. One patient had a confirmed Low Malignant Potential (Borderline) tumor that was considered benign for this analysis.

**FIGURE 6:**
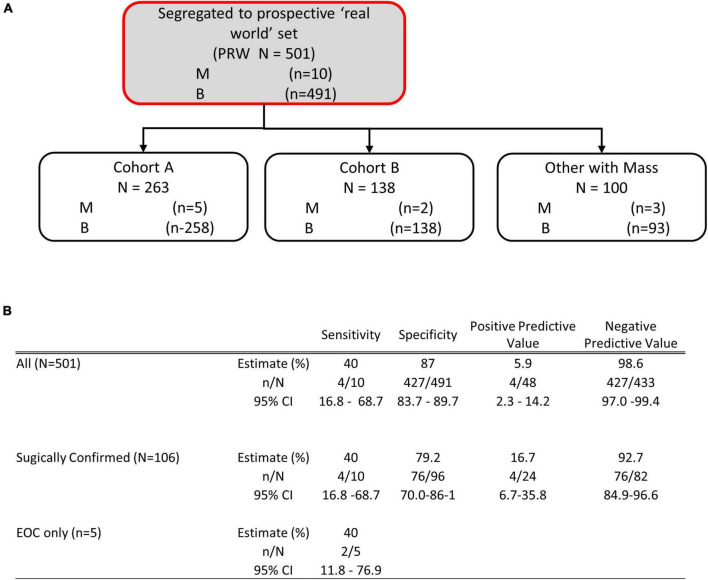
Performance of OVAWatch in the prospective real-world (PRW) study. **(A)** Flow diagram showing patient data represented (shaded, with red outline). The inset Table **(B)** shows the performance for all patients, only those who went to surgery and sensitivity for EOC malignancies.

To examine the performance of OvaWatch in a real-world setting, evaluable patients that did not go to surgery are considered as *histologically benign* in these analyses because they have been followed at least 5 months with TVUS without a reported significant increase in size. This was to approximate the tests’ clinical utility by integrating independent physician assessment into the overall risk assessment. OvaWatch, at a previously validated threshold value of ≥5.0 (13) identified 4 of 10 (sensitivity of 40%) histologically malignant patients as *indeterminate* (Figure 6). Of the 10 total histologically confirmed malignancies, 50% (5) were not epithelial ovarian cancers (EOC), and 50% were considered early-stage. This contrasts with the distribution in a previous analytical validation where EOC represented 80.6% (79/98) malignancies and non-EOC malignancies were 7.6% (6/79) of all malignancies (13). Early-stage cancers comprised 26 of the 79 malignancies. The false positive rate was 12.8% (64/501) when including patients who did not go to surgery, compared with 15.2% (304/2,000) in the published validation report (13).

The study protocols had physicians stratify patients into cohorts based on whether the patient showed physical symptoms (e.g., pain, bloating, unexplained weight loss, frequent urination) and imaging (TVUS or CT) confirmation of an adnexal mass (Cohort A) or showed no physical symptoms but a mass was present by imaging. For this analysis, we grouped cases from Cohort C with the cases where the cohort was not indicated by the physician into a single “Other, with Mass” cohort. Table 3 summarizes the performance of OvaWatch at a score threshold of ≥5.0 in the cohorts for identifying histologically malignant patients as indeterminate. There were differences in sensitivity among these cohorts, but small sample sizes warrant against any comparisons. NPV was above 98% for these cohorts.

**TABLE 3:** Performance of OVAWatch stratified by symptom cohorts.

		Sensitivity	Specificity	Positive predictive value	Negative predictive value
Cohort A (*N* = 263)	Estimate (%)	60	88.4	9.1	99.1
	*n*/*N*	3/5	228/258	3/33	228/230
	95% CI	23.1–88.2	83.9–91.2	3.1–23.6	96.9–99.8
Cohort B (*N* = 138)	Estimate (%)	0.0	89.0	0.0	98.4
	*n*/*N*	0/2	121/136	0/15	121/123
	95% CI	0.0–65.8	82.6–93.2	0.0–20.4	94.3–99.6
Other	Estimate (%)	33.3	80.4	5.0	98.4
With mass (*N* = 100)	*n*/*N*	1/3	78/97	1/19	78/80
	95% CI	6.1–79.2	71.4–87.1	9.0–23.6	91.3–99.3

**FIGURE 7:**
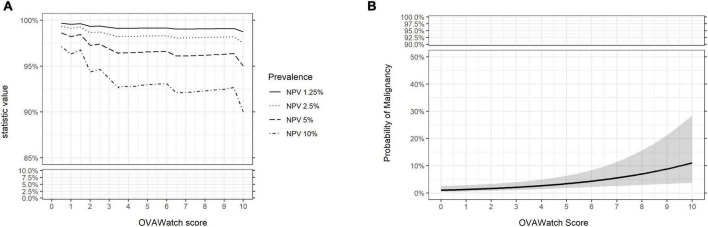
Predicted performance of OVAWatch derived from the prospective real-world (PRW) dataset. **(A)** NPV is plotted as a function of OVAWatch cut-off score. Individual lines represent NPV over OVAWatch score cut-off by predicted prevalences from 1.25–10%. Note the *y*-axis break to emphasize the effects of prevalence on NPV. **(B)** Logistic regression of the probability of malignancy as a function of OVAWatch score.

The performance characteristics of OvaWatch were analyzed across the range of threshold scores to evaluate the stability of performance as a function of thresholds and prevalence. The NPV and PPV were calculated for estimated prevalence between 1.25 to 10% using the formulae presented in the “2. Materials and methods.” The results are presented graphically in Figure 7. As expected, specificity and PPV increased as OvaWatch scores increased, and sensitivity and NPV increased as scores decreased. NPV remained stable across the spectrum of OvaWatch scores. Comparable performance values at projected prevalence of 1.25, 2.5, 5.0, and 10.0% are also shown in tabular form in the Supplementary material.

Additional details regarding the surgical pathology-identified malignancies are presented in Table 4, to further understand the factors contributing to the misclassification of the malignancies. Misclassification of the malignant cases by OvaWatch was not associated with any of the features presented in Table 4.

**TABLE 4:** OVAWatch score in prospective real-world (PRW) patients with malignancies.

Subject	Study cohort	Menopausal status	Age	Type	Histological subtype	Stage	OVAWatch score
PRW1	A	Pre-menopausal	53	Other	Mucinous, intestinal origin	NS*	9.5
PRW2	A	Pre-menopausal	42	Other	Leiomyosarcoma	NS	0.5
PRW3	A	Pre-menopausal	45	EOC	Endometrioid	NS	1.5
PRW4	A	Pre-menopausal	55	EOC	Mixed	II	9.5
PRW5	A	Post-menopausal	53	Other	Granulosa Cell	II	6.0
PRW6	B	Post-menopausal	80	EOC	Epithelial Carcinosarcoma	NS	2.5
PRW7	B	Post-menopausal	54	Other	Granulosa Cell	NS	1.5
PRW8	Other	Post-menopausal	45	EOC	Endometrioid	NS	9.5
PRW9	Other	Post-menopausal	65	EOC	Serous	I	3.0
PRW10	Other	Pre-menopausal	21	Other	Sertoli-Leydig	I	0.0

The shaded cases are misclassified as low risk by OVAWatch score. NS, Not Staged. *NS, Not Staged.

### 3.3. Performance of OvaWatch in an independent high prevalence data set

Because NPV and PPV are prevalence-dependent, we wanted to evaluate how OvaWatch might perform in a clinical context where prevalence is variable. To this end, we addressed the OvaWatch performance characteristics in a high-prevalence population by assembling a data set of independent prospective specimens and specimens of known pathology obtained from commercial sources.

The performance of the independent validation cohort is summarized in Table 5. This cohort was selected from early clinical trial results and supplemented with serum samples from patients with surgical pathology-confirmed malignancies with the goal of producing a simulated high prevalence data set. The prevalence in this data set was 45.8% (38/83). In this high-prevalence cohort (41%), OvaWatch showed 83.3% sensitivity (95% CI: 69.6–92.6%) and 90.2% (CI of 85.2 to 98.8%) specificity over all samples. The NPV was 87.8% (95% CI: 75.8–94.3%).

**TABLE 5:** Performance of OVAWatch in a high-prevalence independent data set.

		Sensitivity	Specificity	Positive predictive value	Negative predictive value
All (*N* = 83)	Estimate (%)	84.2	95.6	94.1	87.8
	*n*/*N*	32/38	43/45	34/34	43/49
	95% CI	69.6–92.6	85.2–98.8	80.9–98.4	75.8–94.3

**FIGURE 8:**
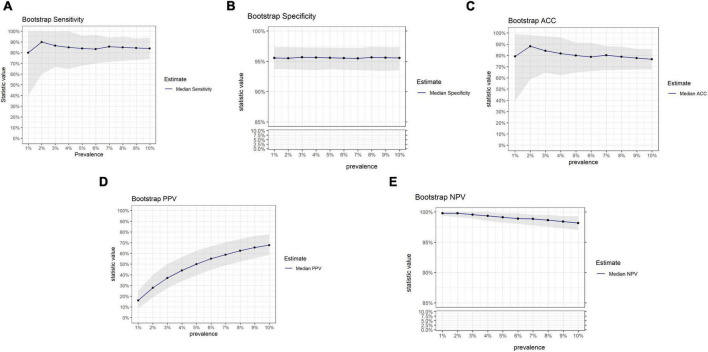
Results of bootstrap analysis to evaluate the effects of prevalence on OVAWatch performance estimates and variability. Each statistic was estimated over 5,000 bootstrap samples at prevalence of 1–10%. The blue line represents the median estimated statistic, the gray band is the 2.5–97.5 percentile of the distributions. Bootstrap estimates are shown for sensitivity **(A)**, specificity **(B)**, Accuracy **(C)**, PPV **(D)**, and NPV **(E)**. Note *y*-axis breaks on panels **(B,E)** to emphasize prevalence dependent changes.

The influence of prevalence on OvaWatch performance from this data set was also examined using bootstrap analysis, simulating sets with increasing prevalence (approximately 1–10%) but similar in size to the PRW data set (*N* = 501). The results are presented in Figure 8. These simulations showed that sensitivity and PPV were most susceptible to prevalence effects. PPV varied in magnitude over prevalence, as expected. Sensitivity showed consistent median level but much larger variance at lower prevalence. NPV and specificity did not vary more than 10% in magnitude over this range.

## 4. Discussion

Many adnexal masses discovered on initial clinical examination can be managed conservatively due to intrinsically low risk of malignancy (2–4). Unnecessary surgical intervention can result in possible surgical complications, loss of productivity, and increased costs to patients (20, 21). Although several tools exist to assess the need for surgical management of adnexal masses suspected to be malignant (12, 14, 22), there have not been effective biomarker-based tests to aid the *clinical management* of women with adnexal mass that is suspected to be benign, and thus nothing to specifically guide conservative management of a mass.

The proposed intended use of OvaWatch is as a non-invasive test to assess the risk of ovarian cancer for women with adnexal masses evaluated by initial clinical assessment as indeterminate or benign. An effective biomarker-based test would need to have the following properties (1) a high NPV for ruling out malignancy when the result is low risk, which would be most of the cases in this intended use group (2) a good sensitivity to not miss a possible malignancy that physicians would otherwise miss using other assessment methods (3) reasonable specificity so as not to place benign masses into a high-risk category. The results presented here supports the fact that OvaWatch achieves these design goals when properly integrated with current clinical practice.

In the PRW sample set, OvaWatch at a threshold score of 5.0 had a NPV of 92.7% for surgically confirmed samples and 98.6% for all samples; these values are within the limits of previous studies (13). NPV was consistent across the cohorts. This validates a role for this test in confirming a benign. The sensitivity of the test (40%, 95% CI: 16.8–68.7%) was much lower in the PRW data set than in the retrospective validation report (13) and the MIAB data set presented here (sensitivity of 81.8%, (95% CI 61.5–92.7%), though the difference was not statistically significant due broad overlapping confidence intervals. This lower sensitivity needs to be acknowledged because it is contrary to the application as a Rule-Out test and was generally poor at the 5.0 threshold value across the cohorts. Our bootstrap investigations suggest that at this low prevalence, however, low sensitivity estimates can be a result of sampling, even where the true population sensitivity may be high. NPV, however, was more stable across the prevalence range in the bootstrap study and will be high in most low-prevalence situations due to the nature of the calculation. This favors a rule-out test where the expected prevalence of malignancy in women presenting with a mass is reportedly less than 10%. Simple adnexal masses have very low risks of malignancy (0–1%) and in masses that are indeterminate by ultrasonography, the incidence is less than 5% (6, 7, 23).

Another contribution to low sensitivity in the PRW may be from the distribution of types and stages of malignancies. Several unusual malignancies were discovered in the PRW study; two Sertoli-Leydig (SLCT) tumors, two granulosa cell tumors (GCT), and one presumed uterine leiomyosarcoma. Clinical data revealed the leiomyosarcoma was diagnosed on a true cut biopsy of a pelvic mass. This most likely was the uterus. At ultrasound examination, most GCTs are large multilocular-solid masses or solid tumors. Tumor markers with this clinical presentation would include inhibin levels, Antimüllerian hormone, or Müllerian-inhibiting substance (24). Sertoli-Leydig cell tumors makeup <0.5% of all ovarian tumors and are benign or malignant, androgen-secreting tumors. They are unilateral and contain solid elements. Patients with Sertoli-Leydig cell tumors often present with masculinization. Testosterone and estrogen levels are appropriate markers (25). These rare tumor types should be suspected on clinical grounds and appropriate tumor markers drawn. Additionally, and as expected for benign mass management data set, a higher percentage of the masses were early stage (50%) as compared to our validation set (13) and published studies of higher risk patients (12, 14). Serial monitoring may increase the frequency of early detection in these patients (26).

Additionally, the study did not address the differential diagnosis of adnexal mass suspicious for adnexal torsion. In fact, the most common ovarian pathologies found in adolescents with adnexal torsion are benign ovarian cysts and teratomas (27, 28). Torsion of malignant ovarian masses is not commonly found. It is important to note that there are no clinical or imaging criteria sufficient to confirm the pre-operative diagnosis of adnexal torsion, and Doppler flow alone should not guide clinical decision making. In this regard, OvaWatch test should not be used to evaluate adnexal mass suspicious for adnexal torsion. Surgical diagnosis is required for adnexal torsion and appropriate surgical management is recommended (29, 30).

The role of the physician in the initial triage of adnexal mass was not systematically investigated in these prospective studies but is likely to play a role in overall diagnostic accuracy for adnexal mass risk of malignancy. We did not collect information on clinical covariates that influenced a decision for surgery (suspicion of malignancy, symptoms, patient comorbidities) and cannot assume all surgical patients were presumed malignant. However, it is highly likely that those patients that did not go to surgery immediately following the initial draw were presumed to be benign by the physician, and this is supported by the high specificity in physician assessment alone (7–9). It follows that the specificity of OvaWatch measured for this population closely approximates the actual specificity in the absence of all surgical information, and that the resulting NPV is also representative. It would be useful to evaluate OvaWatch in conjunction with consensus imaging and triage tools (31) with the intent to increase pre-operative diagnostic accuracy.

In published data on MIA and MIA2G (10, 12, 14), the collection of an independent physician assessment permitted authors to demonstrate that the “OR” combination of biomarker and physician assessment yielded improved sensitivity for detecting malignancy. However, this reduced the specificity of the test. We would not suggest a similar algorithm for assessing potentially benign masses, as it would tend to over-diagnose patients as at risk, but data from our retrospective studies (Figure 3 and Supplementary Figure 1) indicate more favorable outcomes when physician and biomarker assessments are combined. Physicians were generally good at assessing a benign mass, even in this population of patients where all enrolled patients were scheduled for surgery. For instance, Coleman et al. (10) showed in data from the Ova500 study–which contained specimens later used to either develop or validate OvaWatch–that physician assessment alone had a specificity of 92.8% (95% CI: 89.8–94.9%) for all evaluable subjects. Although the sensitivity in that study was 73.9% (95% CI: 64.1–81.8%), the addition of MIA2G increased the sensitivity to 93.5%. In our stratified prospective MIAB dataset, OvaWatch was able to identify 18/22 malignancies as an elevated risk that physicians assessed to be benign in the retrospective set, while it identified all the malignancies that physicians also assessed as malignant. It should be noted that OvaWatch also identified 180 benign patients as indeterminate (false positive rate of 12.6%), and this suggests that OvaWatch might benefit from incorporation into clinical algorithms, or further neural network training against false positives to ameliorate these classification errors.

The performance of OvaWatch across the scores (Supplementary Table 1) indicated the threshold OvaWatch value of ≥5.0 for indeterminate results did not result in the highest performance for the PRW data set. Lower values of OvaWatch in this set would result in higher sensitivity without much of a drop in specificity. The low prevalence may have been an influence on sensitivity. In the PRW data set with only 10 malignancies, the cutoff at 5.0 had an NPV of over 99%. At an OvaWatch score of 2.5, the sensitivity would be 60%, and the specificity 79%, without impacting NPV. The performance of OvaWatch across a range of cutoffs and prevalence, and the stability of NPV (Figure 4B) suggest that physicians should be able to interpret a risk level relevant to the clinical and pathological parameters of the patient or cohort they are evaluating. A physician may choose a more conservative approach in patients with comorbidities and patients who may need to or want to delay surgery due to personal or professional reasons. In a higher prevalence population, the PPV will increase, and physicians may choose to use a cutoff that supports a higher PPV and specificity to identify patients at risk for ovarian malignancy. In such scenarios, as supported by these diverse data sets, the NPV provides confidence in the high probability of benign adnexal mass. This is also reflected in the probability of malignancy as a function of the OvaWatch score. As Figures 4A, 6B show, the probability of an abnormal (malignant?) mass as a function of the OvaWatch score is not significantly different between the MIAB and PRW studies. From the logistic regression, the upper limit of the 95% CI of the probability of malignancy was 3.7% for the MIAB study and 6.4% for the PRW study at a threshold value of 5.0. Importantly, such personalized approach in interpreting OvaWatch result supporting physicians’ decision-making process, including the importance of fertility-sparing approach and reproductive outcomes as one of the clinical consequences of patient management (32–34).

It is important to note that prospective data set is limited in size. The low likelihood of finding malignancies in patients with incidentally discovered and mostly simple cystic adnexal masses and the rare nature of ovarian cancer impacted the prevalence and likely the accuracy of performance metrics. Information on imaging and physician impression of the masses was also not recorded frequently enough to allow meaningful stratification on other diagnostic factors and this may hide interesting interactions in the data. Even though the study did not collect specific information of benign masses, the performance of OvaWatch might support the evaluation of adnexal mass suspicious as endometriosis cysts versus a mass arising from an endometrioid ovarian cancer or metastatic endometrial cancer to the ovary (32, 35–39) through the testing result of low probability of malignancy versus indeterminate.

We also acknowledge that OvaWatch was designed on data that was initially collected to address a higher risk population. The algorithm was developed and validated on a highly diverse cohort obtained by merging several studies–mostly retrospective–with data collected from patients who were confirmed to have an adnexal mass and scheduled for surgery at the time of diagnosis. The influence of the higher risk patients on the biomarker values in the train/test sets have led to low sensitivity in the prospective populations, collected from women at lower risk. However, it is noteworthy how consistent the performance has been over these seemingly disparate data sets. Finally, not all patients were followed up with surgery, and so did not receive the “gold standard” diagnostic outcome. The assumption of benign mass for many in this study is based on hypotheses about the low rate of progression of masses over time (8, 40) and physician assessment.

## Data availability statement

The datasets presented in this article are not readily available because the data are proprietary to Aspira Women’s Health. Requests to access the datasets should be directed to RP, rphan@aspirawh.com.

## Ethics statement

The studies involving human participants were reviewed and approved by Advarra. The patients/participants provided their written informed consent to participate in this study.

## Author contributions

GR, CD, RB, DU, TP, and RP: conception and design. GR, CD, and HF: provision of study materials or patients. RB, DU, and TP: collection and assembly of data. DU, SG, RB, and TP: data analysis and interpretation. All authors contributed to the manuscript authoring and approval.
