# Supplementary material for: Validation of a deep neural network-based algorithm supporting clinical management of adnexal mass
Source: Front Med (Lausanne). 2023 Jan 23;10:1102437. doi: 10.3389/fmed.2023.1102437 (PMC9900123; doi:10.3389/fmed.2023.1102437)
Supplement: Supplementary file 1 [file Data_Sheet_1.docx]

Supplementary Material

# Supplementary Tables

## Supplementary Table 1

Sensitivity, specificity, negative predictive value (NPV) and positive predictive value (PPV) as a function of OVAWatch score in data from the MIAB dataset.

| OVAWatch Score | Sensitivity | Specificity | NPV | PPV |
| --- | --- | --- | --- | --- |
| 0 | 100.0% | 0.0% | NA | 1.5% |
| 0.5 | 95.5% | 47.0% | 99.9% | 2.7% |
| 1 | 90.9% | 64.4% | 99.8% | 3.8% |
| 1.5 | 86.4% | 70.4% | 99.7% | 4.3% |
| 2 | 81.8% | 76.3% | 99.6% | 5.0% |
| 2.5 | 81.8% | 78.9% | 99.6% | 5.6% |
| 3 | 81.8% | 81.3% | 99.7% | 6.3% |
| 3.5 | 81.8% | 82.9% | 99.7% | 6.9% |
| 4 | 81.8% | 84.5% | 99.7% | 7.5% |
| 4.5 | 81.8% | 86.0% | 99.7% | 8.2% |
| 5 | 81.8% | 87.4% | 99.7% | 9.1% |
| 5.5 | 81.8% | 88.3% | 99.7% | 9.7% |
| 6 | 81.8% | 89.2% | 99.7% | 10.5% |
| 6.5 | 81.8% | 90.1% | 99.7% | 11.3% |
| 7 | 77.3% | 91.2% | 99.6% | 11.9% |
| 7.5 | 77.3% | 92.0% | 99.6% | 12.9% |
| 8 | 77.3% | 92.7% | 99.6% | 14.0% |
| 8.5 | 72.7% | 94.2% | 99.6% | 16.2% |
| 9 | 68.2% | 95.7% | 99.5% | 19.7% |
| 9.5 | 59.1% | 97.8% | 99.4% | 28.9% |
| 10 | 0.0% | 100.0% | 98.5% | NA |

## Supplementary Table 2

Sensitivity, specificity, negative predictive value (NPV) and positive predictive value (PPV) estimated from prevalence as a function of OVAWatch score in data from the PRW dataset.

|  |  |  | 1.25% | | 2.50% | | 5% | | 10% | |
| --- | --- | --- | --- | --- | --- | --- | --- | --- | --- | --- |
| OVAWatch Score | Sensitivity | Specificity | NPV | PPV | NPV | PPV | NPV | PPV | NPV | PPV |
| 0 | 100.0% | 0.0% | NA | 1.3% | NA | 2.5% | NA | 5.0% | NA | 10.0% |
| 0.5 | 90.0% | 37.7% | 99.7% | 1.8% | 99.3% | 3.6% | 98.6% | 7.1% | 97.1% | 13.8% |
| 1 | 80.0% | 58.0% | 99.6% | 2.4% | 99.1% | 4.7% | 98.2% | 9.1% | 96.3% | 17.5% |
| 1.5 | 80.0% | 66.6% | 99.6% | 2.9% | 99.2% | 5.8% | 98.4% | 11.2% | 96.8% | 21.0% |
| 2 | 60.0% | 74.5% | 99.3% | 2.9% | 98.6% | 5.7% | 97.3% | 11.0% | 94.4% | 20.8% |
| 2.5 | 60.0% | 78.8% | 99.4% | 3.5% | 98.7% | 6.8% | 97.4% | 13.0% | 94.7% | 23.9% |
| 3 | 50.0% | 81.9% | 99.2% | 3.4% | 98.5% | 6.6% | 96.9% | 12.7% | 93.6% | 23.5% |
| 3.5 | 40.0% | 83.7% | 99.1% | 3.0% | 98.2% | 5.9% | 96.4% | 11.4% | 92.6% | 21.4% |
| 3.5 | 40.0% | 85.3% | 99.1% | 3.3% | 98.2% | 6.5% | 96.4% | 12.6% | 92.8% | 23.3% |
| 4.5 | 40.0% | 86.2% | 99.1% | 3.5% | 98.2% | 6.9% | 96.5% | 13.2% | 92.8% | 24.3% |
| 4.5 | 40.0% | 87.0% | 99.1% | 3.7% | 98.3% | 7.3% | 96.5% | 13.9% | 92.9% | 25.4% |
| 5 | 40.0% | 88.2% | 99.1% | 4.1% | 98.3% | 8.0% | 96.5% | 15.1% | 93.0% | 27.3% |
| 6 | 40.0% | 89.6% | 99.2% | 4.6% | 98.3% | 9.0% | 96.6% | 16.9% | 93.1% | 30.0% |
| 6.5 | 30.0% | 90.6% | 99.0% | 3.9% | 98.1% | 7.6% | 96.1% | 14.4% | 92.1% | 26.2% |
| 7 | 30.0% | 91.0% | 99.0% | 4.1% | 98.1% | 7.9% | 96.1% | 15.0% | 92.1% | 27.1% |
| 7.5 | 30.0% | 92.1% | 99.0% | 4.6% | 98.1% | 8.8% | 96.2% | 16.6% | 92.2% | 29.6% |
| 8 | 30.0% | 93.1% | 99.1% | 5.2% | 98.1% | 10.0% | 96.2% | 18.6% | 92.3% | 32.5% |
| 8.5 | 30.0% | 94.5% | 99.1% | 6.5% | 98.1% | 12.3% | 96.2% | 22.3% | 92.4% | 37.7% |
| 9 | 30.0% | 95.5% | 99.1% | 7.8% | 98.2% | 14.7% | 96.3% | 26.1% | 92.5% | 42.7% |
| 9.5 | 30.0% | 98.2% | 99.1% | 17.2% | 98.2% | 29.6% | 96.4% | 46.3% | 92.7% | 64.5% |
| 10 | 0.0% | 100.0% | 98.8% | NA | 97.5% | NA | 95.0% | NA | 90.0% | NA |

## Supplementary Figures


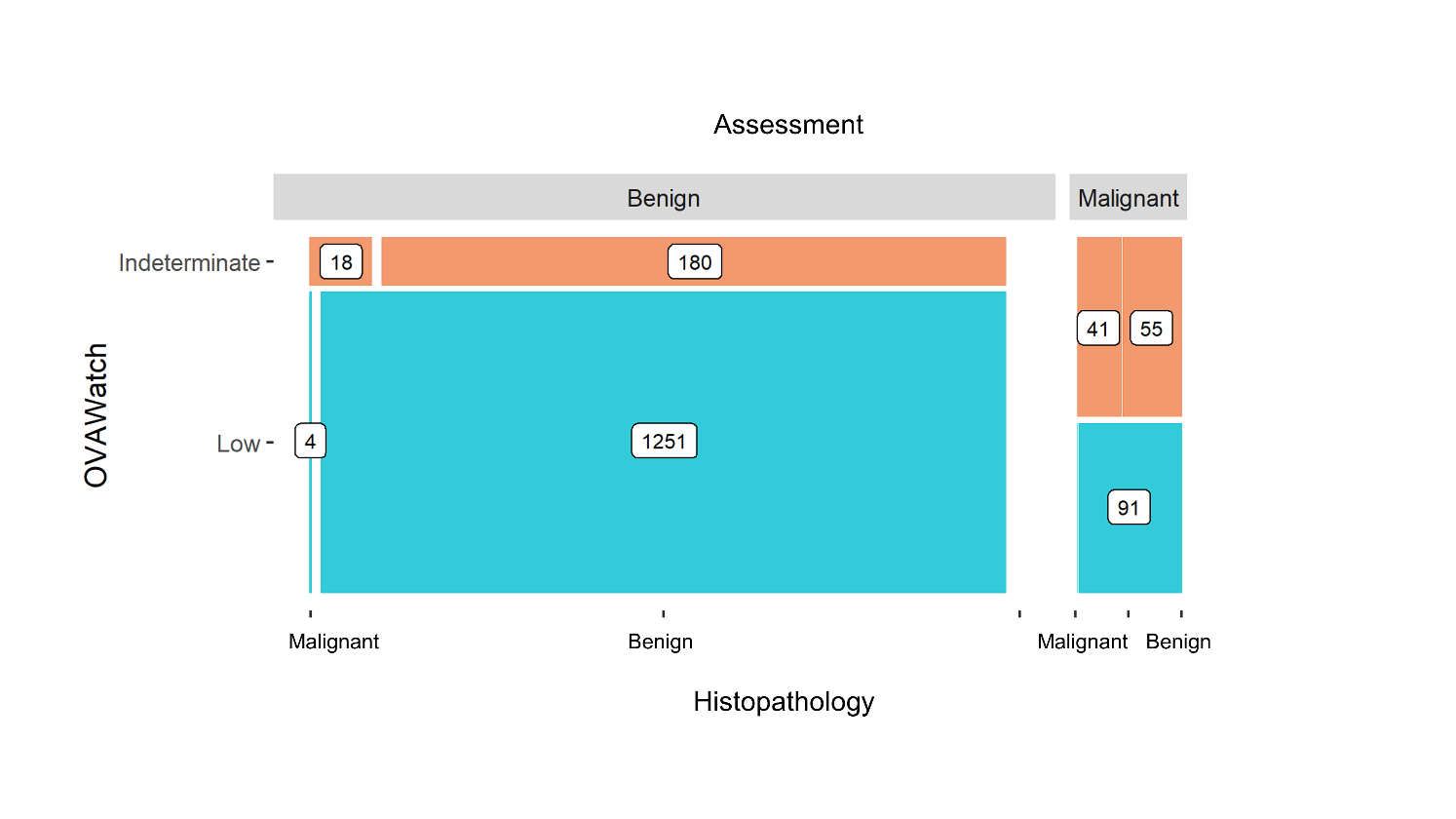


**Supplementary Figure 1.** Matrix plot of the retrospective samples from the previous validation that were determined to be either Assessment Benign or Assessment Malignant by physicians prior to surgical confirmation of pathology. The matrix plot shows the performance of OVAWatch in classifying masses as Indeterminate (orange) or Low Probability of Malignancy (blue) that were later determined to be Histopathology malignant (Malignant) or histologically benign (Benign). Numbers in tiles represent the number of patients in each subcategory. Note that there were no patients who were assessment malignant, histopathology malignant and OVAWatch low.
